# Production of sterile mono-sex triploid yellow drum (*Nibea albiflora*): genotypic females and sex-reversed phenotypic males with emphasis on utilization as surrogate broodstock

**DOI:** 10.1007/s10695-023-01256-8

**Published:** 2023-10-25

**Authors:** Yang Yang, Lei Lu, Ruiyi Chen, Liechao Yu, Weihua Hu, Dongdong Xu

**Affiliations:** 1 Key Laboratory of Mariculture and Enhancement of Zhejiang Province, Zhejiang Marine Fisheries Research Institute, Zhoushan, 316021 People’s Republic of China; 2 School of Fisheries, Zhejiang Ocean University, Zhoushan, China; 3 School of Marine Science, Ningbo University, Ningbo, China

**Keywords:** Mono-sex, Triploidization, Gametogenesis, Sterile, Abnormal meiosis

## Abstract

Production of sterile mono-sex fish is of great significance for sustainable aquaculture as well as germ cell transplantation. In this study, we aimed to produce mono-sex triploid yellow drum, including genotypic females (XXX female) and sex-reversed phenotypic males (XXX male). Firstly, the mono-female triploids were produced through cold-shock treatment on eggs fertilized with sperm from neo-males. Then, the mono-male triploids were produced by the sex reversal of mono-female triploids with oral administration of letrozole (LZ). We comparatively investigated the growth and gonadal development in the mono-sex triploids. The results showed that the triploids displayed similar growth performance to their diploids throughout their first year, but had impaired gonadosomatic index and gametogenesis. No mature gametes were produced in the triploids during their first spawning season. Meanwhile, we analyzed the process of gametogenesis in the both sex of triploids. Ultrastructure of gametogenesis showed that the germ cells arrested at abnormal metaphase 1 in females, while males had irregular meiotic divisions, variable-sized spermatid and degenerated cells. The expression levels of meiosis-related genes (i.e., *sycp3* and *rec8*) confirmed the abnormal meiosis in the triploids. Furthermore, the gonadal development was also determined by the expression patterns of *vasa*, *dmrt1* and *cyp19a1a*. Abnormal expression of *vasa* mRNA and protein were detected in triploids. High *cyp19a1a* expression levels suggested the sex steroid hormones production might be at least partially functional in triploid females. In addition, high *dmrt1* expression levels confirmed the masculinization and testicular development of sex-reversed triploid males by LZ. Our findings provide an efficient protocol to produce sterile mono-sex triploid yellow drum and provide new insights into the mechanism of gonadal sterility of triploid fish.

## Introduction

Triploidy is a condition of having three sets of chromosomes (3n) in an organism or cell. In aquaculture, inducing triploidy is a commonly-used method to produce sterile fish with improved growth performance and reduced reproductive problems (Tiwary et al. 2004; Maxime 2010). In general, triploid fish are functionally sterile because they cannot produce normal gametes due to the irregular meiotic division of chromosomes. Therefore, triploid fish can avoid the energy consumption for sexual maturation and allocate more energy towards somatic growth. Moreover, sterile triploid fish can minimize the environmental impacts of escaped culture fish and avoid genetic interactions of the wild population.

In aquaculture, the triploidy has been achieved in many fish species, such as rainbow trout (*Oncorhynchus mykiss*) (Lincoln and Scott 1983), blue tilapia (*Oreochromis aureus*) (Mol et al. 1994), crucian carp (*Carassius auratus*) (Zhang et al. 2021) and sea bass (*Dicentrarchus labrax*) (Felip et al. 2001). Most of triploid fish have a growth advantage over diploid fish (Okomoda et al. 2020; Tiwary et al. 2004). However, the growth advantage of triploids is not always apparent (Aydın et al. 2022; do Nascimento et al. 2017; Mol et al. 1994), and some studies have found that triploid fish have greater mortality than diploid fish (Cotter et al. 2000; Koenig et al. 2011). Meanwhile, there are also numerous studies of gonadal development in triploid fish. For instance, impaired gonadal development and abnormal gametogenesis were found in the most of triploid fish (Comai 2005; Golpour et al. 2016; Takeuchi et al. 2018). However, some studies also have found the triploids could produce small amounts of mature gametes (do Nascimento et al. 2017; Hamasaki et al. 2013; Zhang et al. 2021). Therefore, the characteristic of growth and gonadal development of triploid fish is species-specific. On the other hand, many fish species have sexual dimorphic in growth, with female fish would grow faster (Wu and Gui 1999; Bye and Lincoln 1986; Chen et al. 2007) or slower (Beardmore et al. 2001; Farley et al. 2014) than males. Therefore, the production of mono-sex populations of triploid fish is of great significant for improving its yield and provide an effective mean for sustainable aquaculture. Meanwhile, mono-sex triploid fish also have the potential for scientific research, such as serving as surrogate recipients for germ cell transplantation (Okutsu et al. 2007; Yoshizaki et al. 2011).

Yellow drum (*Nibea albiflora*), which has an XY sex determining system, is one of the most commercially valuable Sciaenidae species distributed in East Asia (Sun et al. 2018). Especially in China, this species is economically important for fisher and aquaculture due to its high market demand and nutritional value (Xu et al. 2018; Chen et al. 2017). Yellow drum has a high tolerance to crowding and a wide range of physiological conditions, making it suitable for intensive aquaculture in sea cages along the coastal waters of China (Xu et al. 2018; Tian et al. 2020). Meanwhile, yellow drum also has a sexually dimorphic in growth, with females growing faster and larger than males at the same age (Qin et al. 2019). Previous studies have successfully established a system for producing all-female yellow drum by mating of normal females with phenotypic males (known as neo-males) (Chen et al. 2017; Xu et al. 2018; Qin et al. 2019). However, the studies about the production of mono-sex triploid yellow drum have not reported. Although the gonadal development of triploid Nibe croaker (*Nibea mitsukurii*) has reported in previous work (Takeuchi et al. 2018), the knowledge on the growth and detailed gonadal development of mono-sex triploid yellow drum also remains unclear.

In this study, we aimed to produce mono-sex triploid yellow drum, including genotypic female triploids (XXX female) and sex-reversed phenotypic male triploids (XXX male). Firstly, the mono-female triploids were produced through cold-shock treatment on eggs fertilized with sperm from neo-males. Then, the mono-male triploids were produced by the sex reversal of mono-female triploids with oral administration of letrozole (LZ), an aromatase inhibitor that can induce sex reversal in fish. We also investigated the growth performance, gonadal development, gametogenesis and gene expression of the triploids from 75 to 390 days post hatching (dph), a period that encompasses their first reproductive cycle. To our knowledge, this is the first study on the production and characterization of mono-sex triploid yellow drum.

## Materials and methods

### Broodstock management and gamete collection

At May 2020, yellow drum broodstock were obtained from the research station of Zhejiang Marine Fisheries Research Institute (Xishan Island, City of Zhoushan, China) and reared in 24 m^2^ indoor tank with flow seawater under controlled conditions (a photoperiod of 13 h light vs. 11 h dark and a water temperature of 18–22 ℃). All experiments were approved by the Institutional Animal Care and Use Ethics Committee of the Zhejiang Marine Fisheries Research Institute, China. To induce spawning, the females were intraperitoneally injected with luteinizing hormone releasing hormone (LHRH)-A2 (Ningbo Second Hormone Factory, Ningbo, China) at a dose of 1.5 ug/kg body weight. Thirty-two hours after the hormone injection, ovulated eggs were collected from each female by gently squeezing the abdomens by hands for use in artificial insemination. At the same time, milt was also collected from ripe males (without the injection of exogenous hormone) by pressing their abdomens and subsequently mixed. The eggs and milt were held separately in dry glass bowls and kept at 4 ℃ in the dark prior to use. One mL milt was mixed with approximately 16,000 eggs from each female, and sperm was subsequently activated by adding 100 mL 22 ℃ seawater. Five minutes (min) after fertilization, 500 mL seawater were added again.

### Production of genotypic female and sex-reversal phenotypic male triploid yellow drum

In order to produce genotypic triploid females (XXX female), the eggs were separately obtained from 5 genotypic diploid females of three years-old (XX, female), and fertilized with sperm obtained from 3 diploid neo-males of two years-old (XX, male). After artificial insemination, the fertilized eggs received a cold-shock treatment to suppress second polar body extrusion, according to the protocol optimized by Chen et al. (2017). Briefly, the eggs were maintained in seawater at 3 ℃ for 10 min at 2.5 min after fertilization, and then cultured in 1000-L seed production tank under 21–22 ℃.

**Fig. 1:**
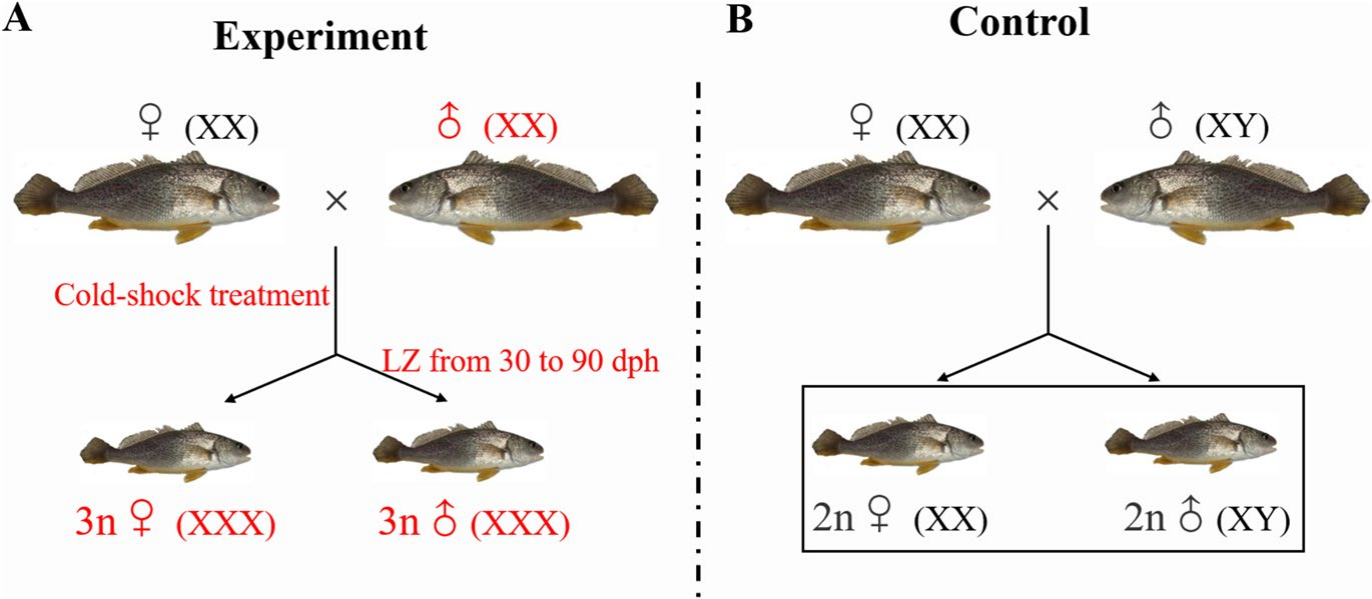
Experiment design for production of mono-sex triploid yellow drum. Genotypic triploid females (3n♀, XXX) were produced by cold-shock treatment (at 3 ℃ for 10 min at 2.5 min after fertilization) on eggs fertilized with sperm from neo-males, and phenotypic triploid males (3n♂, XXX) were produced from sex-reversal of genotypic triploid females by oral administration of letrozole (LZ) from 30 to 90 dph (**A**). The eggs (from 3 genotypic diploid females of three years-old) fertilized using sperm obtained from 3 normal diploid males of two years-old (XY) and treated without cold-shock treatment were used as controls (**B**). LZ, letrozole; dph, days post hatching

At 25 dph, a total of 1200 fish of genotypic triploid female survived. In our previous study, the diploid yellow drum were 100% sex reversed into neo-males by orally administered of 10 mg/kg LZ for 60 days (from 25 to 85 dph) (Qin et al. 2019). Therefore, in this study, a half of genotypic triploid females (approximately 600) were selected and transferred to 3 fiber-reinforced plastic (FRP) tanks (200-L, *n* = 200 fish per tank), and were orally administered a dose of 10 mg/kg LZ from 30 to 90 dph to produce sex-reversed phenotypic triploid males (XXX, male). Briefly, the LZ (Macklin, Shanghai, China) was dissolved in 100% ethanol, and slowly poured onto the commercial feed pellets to make pellets containing dose of 10 mg/kg LZ. On the other hand, the eggs (from 3 genotypic diploid females of three years-old) fertilized using sperm obtained from 3 normal diploid males of two years-old (XY) and treated without cold-shock treatment were used as controls. The experimental design is illustrated in Fig. 1. Larvae and juvenile fish were reared as described in Xu et al. (2018). Briefly, the fish were fed twice-daily (larvae were fed with *Brachionus rotundiformis* and *Artemia salina*, and juveniles were fed with the commercial pellets). At 150 dph, fish of each group was individually marked by injection of different dye into the dorsal fin, then mixed and reared together in 36 m^2^ tank.

At 12 h post fertilization, 200 to 300 eggs at the blastula stage were collected and examined under a stereo microscope. The fertilization rates were calculated by dividing the number of developing embryos by the total number of eggs and expressed as a percentage. The hatching rate were evaluated approximately 24 h after hatching from 200 to 300 fertilized eggs, and determined as the number of morphologically normal larvae divided by the number of fertilized eggs, which was also expressed as a percentage.

### Ploidy determination

In order to determine ploidy of triploids, the relative DNA content of hatched larvae at 1 dph and the number of chromosomes of 5-month-old fish were measured. Hatched larvae obtained from cold-shock treatment and control were cleaned with distilled water twice and placed in 1.5 mL microcentrifuge tubes (5 to 10 larvae per tube) with 400 uL Nuclei Extraction buffer (CyStain UV Precise P, Sysmex, Japan). After shredding treatment, 1.6 mL staining buffer with 4,6-diamidino-2-phenyl-indole (DAPI) was added to each of the microcentrifuge tubes. Then, the mixture was filtered by a 40 um filter and placed in a new test tube. Finally, fluorescence intensity of DAPI-stained cells was measured by a flow cytometer (CyFlow Ploidy Analyzer, Sysmex, Japan). The operation and measurements were repeated 5 times.

Five-month-old cold-shock treated and control fish were intraperitoneally injected with 5 mL/kg^−1^ (body weight) of 0.1% phytoagglutinin and 5 mL/kg^−1^ (body weight) of 0.5% colchicine. Three hours after injection, the kidneys were removed into 0.075 mol/L KCL solution, minced to dissociate the cells, and held for 30 min at room temperature. Then the supernatant liquid was replaced with freshly prepared Carnoy’s solution (methanol/glacial acetic acid = 3:1) for 90 min at 4 ℃, and dropped onto slides. The chromosomes were stained with 15% Giemsa solution and photographed under light microscope (model BX-51N-34FL, Olympus, Japan). More than 30 metaphases were examined (10 fish were examined for both cold-shock and control groups).

### Sampling and growth performances analysis

In order to examine the growth performance and gonadal development, triploids (genotypic females and sex-reversal phenotypic males) and diploids were sampled at 75, 90, 120, 150, 180, 210, 270, 300, 360 and 390 dph. Thirty randomly selected fish were sampled for each group. At the same time, the body length (BL) and weight (BW) of each fish were recorded.

### Gonadal histology, sex ratios and gonadosomatic index (GSI)

Randomly selected six fish of each group at each time for gonadal histology. After anesthetization, each fish was dissected, then the left gonad was fixed in Bouin’s solution for histological analysis and immunohistochemistry, and the right gonad was fixed in RNAwait (Biosharp, Guangzhou, China) and then stored at -80 ℃ for Quantitative real-time PCR (qPCR). For histological analysis, the fixed gonads were embedded in paraffin, cut into 5-um-thick sections, and stained with hematoxylin and eosin (HE). Ovaries were confirmed by the presence of an ovarian cavity in the transverse section of the gonads. Testes were identified by the presence of sperm duct and spermatocytes in the section of the gonads. Sections were examined and photographed using a light microscopy (model BX-51N-34FL; Olympus, Japan).

At 90 and 120 dph, a total of 120 fish including 40 genotypic triploid females, 40 sex-reversal phenotypic triploid males and 40 normal diploids were used in the sex ratios analysis. Besides, the GSI (gonad weight/body weight) were calculated at 270 and 300 dph.

### Transmission electron microscopy (TEM) analysis

In order to further analyze the ultrastructure of germ cells, 12 individuals from both triploid females and males at 360 and 390 dph were selected for TEM assay. The dissected gonads were cut into fragments (< 1 mm) and immersed them by 2.5% glutaraldehyde in 1 × phosphate buffered saline (1 × PBS) (pH 7.4) for 24 h at 4 ℃. Then the gonads were washed with 1 × PBS, fixed with 2% osmium tetroxide (OsO4) for 4 h at 4 ℃ and dehydrated by using a graded series of ethanol (30, 50, 75, 95, 100 and 100%) for 10–20 min each. They were then embedded in Epon-Araldite for subsequent ultrathin sectioning. Ultrathin Sects. (75–80 nm) were cut using the Ultracut E ultramicrotome, stained with uranyless EM stain, counterstained them with 1% lead citrate, and examined under Zeiss Libra 120 transmission electron microscope (Zeiss, Oberkochen, Germany).

### Quantitative real-time PCR (qPCR)

At 270, 300, 360 and 390 dph, the expression patterns for gonad-specific genes including *vasa* and *cyp19a1a* in ovary, and *vasa* and *dmrt1* in testis were examined via qPCR. At the same time, the relative abundances of meiosis-related genes (*rec8* and *sycp3*) in gonads of both sexes at 300 and 360 dph were also examined via qPCR. The specific primers of the genes were designed using Primer Premier 5.0 and listed in Table 1. *β-actin* and *18 s* was chosen as the reference genes. Total RNA was extracted from ovaries and testes using the RNA extraction kit (Solarbio, China). The first-strand cDNA was synthesized using a Transcript First-strand cDNA Synthesis Kit (TranStart, China). The qPCR amplifications were performed using 2 × SYBY Premix Ex TapTM II (Takara, China) according to the manufacturer’s instructions. PCR amplification was performed using a reaction volume of 20 μL, with 6.8 μL ddH_2_O, 0.4 μL forward and reverse primers (10 μmol/L), 2 μL cDNA, 0.4 μL ROX Reference Dye I and 10 μL 2 × SYBY Premix Ex TapTM II. The PCR reaction was performed under the following conditions: 30 s at 95 ℃; 5 s at 95 ℃ and 30 s at 60 ℃ for 33 cycles; finally 15 s at 95 ℃, 30 s at 60 ℃, and 15 s at 95 ℃ was used for the dissociation stage. Each assay included a no-reverse transcriptase and a no-template control. The relative gene expression levels were analyzed using the 2^−ΔΔCT^ method.

**Table 1:** The specific primers of genes used in this study

Gene	Forward (5’-3’)	Reverse (5’-3’)
*vasa*	GTGTGCGAAAACGGCTTTAG	CCATCGCTTCCATCCTTATTTTC
*cyp19a1a*	TACCTGTGAACGAGAAAGAGC	TCTTGTGCCTCTGGTGAATC
*dmrt1*	AAACCACGGCTATGTCTCAC	CGTCCTTCCATAGAGTACAAACAG
*sycp3*	GCCACGCTGCAGAAGAAGAT	GCATGGACTGCAGGGACTTC
*rec8*	GAGGAGGCCCAGCTACCAAT	CTCTGCAGGCTGGAGGAGAT
*β-actin*	CCTCCCTGGAGAAGAGCTATGAG	CGCACTTCATGATGCTGTTGTAG
*18 s*	CTGCACGGACAGAAACTCAA	CTCTTTAGCCCCCTCTGCTT

### Immunohistochemistry

The localization of Vasa in gonads of both the triploids and diploids at 360 dph was detected using Vasa antibody (ab209710, Abcam, UK). Specifically, the gonadal samples fixed in Bouin’s solution were embedded in paraffin wax, and then sliced into 5–7 um serial sections. The tissue sections were rehydrated with PBS, digested with proteinase K (10 ug/mL) for 30 min, and then fixed in 4% PFA for 20 min, washed with PBST for 5 min four times. Subsequently the samples were incubated with Vasa antibody at a 1: 1000 dilution at 4 ℃ overnight, then washed in PBST and incubated with the secondary antibody at a 1:2000 dilution for 30 min at room temperature. Finally, the samples were stained with DAPI (E607303, Sangon, China) for 10 min, and washed in PBST. Fluorescence images were captured with a fluorescence microscope (model BX-51N-34FL, Olympus, Japan).

### Statistical analysis

All data were presented as means ± standard error of the mean (SEM) using GraphPad Prism 9.0 software. Significant differences in BL and BW were determined using one-way ANOVA followed by the Kruskal-Waillis multiple comparisons test (*P* < 0.05). Significant difference in GSI, gene expression, fertilization and hatching rates were determined using the Student’s t-test (Mann–Whitney test) (*P* < 0.05).

## Results

### Fertilization, hatching and survival rates

The fertilization rates of the cold-shock treated and control embryos were 63.64 ± 6.56% and 84.25 ± 7.63%, respectively. The hatching rates of the cold-shock treated and control embryos were 22.32 ± 3.73% and 70.03 ± 3.03%, respectively. Obviously, the fertilization and hatching rates were significantly affected by the cold-shock treatment in the present study. From 30 dph, the survival rates of sex-reversed triploid males and genotypic triploid females were high (> 90%), and there was no significant difference (*P* > 0.05).

### Ploidy determination in yellow drum

**Fig. 2:**
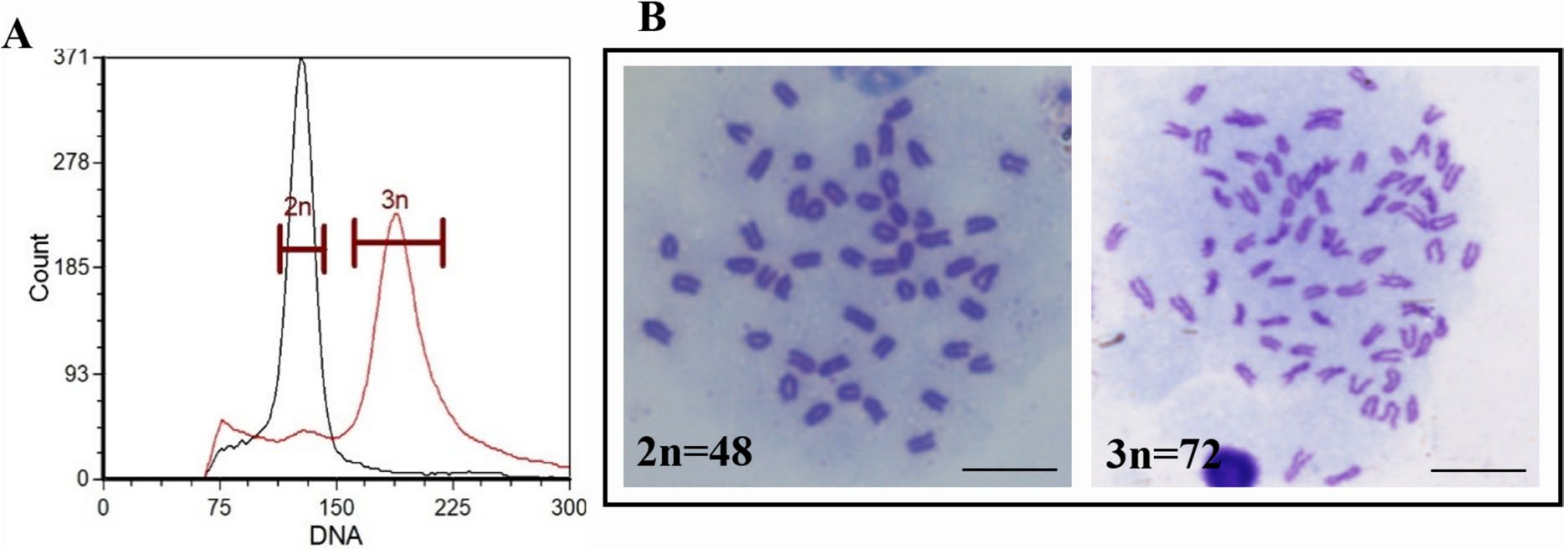
Ploidy determination in triploid yellow drum. The relative DNA content of fin cells from diploid and triploid yellow drum. The horizontal coordinate indicates the DNA content, and the vertical coordinate indicates the cell counts. The DNA contents of diploid and triploid yellow drum were 124 and 186, respectively (**A**). The chromosomes numbers of diploid (2n) and triploid (3n) yellow drum were 48 and 72, respectively (**B**). Scale bars = 10 μm

As shown in Fig. 2A, the DNA content of diploid and triploid yellow drum was 124 and 186, respectively. The DNA content of the triploid fish was 1.5 times than that of the diploid. Microscopic observation of metaphase plates showed that the chromosome number (3n = 72) of triploid fish was 1.5 times larger than that of diploid fish (2n = 48) (Fig. 2B). Triploids had a whole set of chromosomes more than diploid fish.

### Sex ratios of triploid and diploid yellow drum

The sex ratios were confirmed by histological examinations and shown in Table 2. The sex ratios were 100% female in genotypic triploid females and 100% male in sex-reversal phenotypic triploid males. However, the sex ratios were close to 1:1 (55% and 60% male) in normal diploids.

**Table 2:** The sex ratios of the normal diploids, genotypic female triploids (3n♀) and sex-reversed male triploids (3n♂) of yellow drum at 90 and 120 days post hatching (dph)

Date/dph	Groups	Male ratio	Female ratio
	2n(*n* = 20)	55%	45%
90	3n♂(*n* = 20)	100%	0
	3n♀(*n* = 20)	0	100%
	2n(*n* = 20)	60%	40%
120	3n♂(*n* = 20)	100%	0
	3n♀(*n* = 20)	0	100%

### Growth performances in triploid yellow drum

**Fig. 3:**
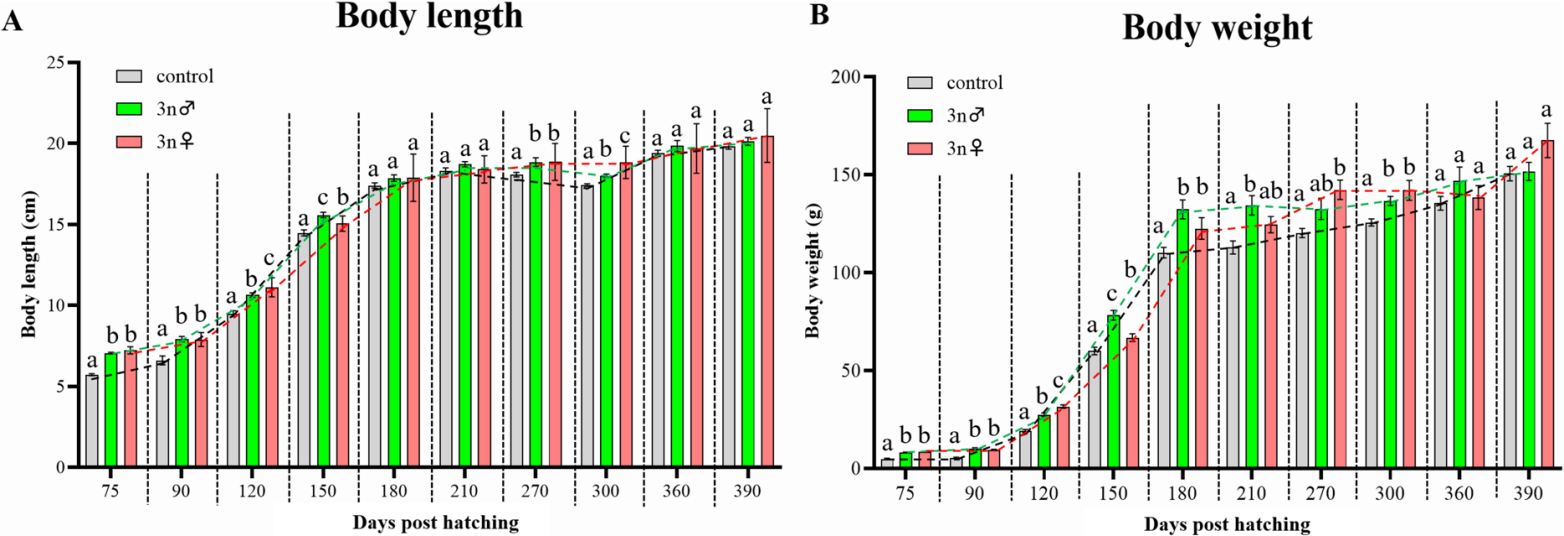
Body length and body weight among diploids (2n), genotypic female triploids (3n♀) and sex-reversed male triploids (3n♂) at 75, 90, 120, 150, 180, 210, 300, 360, and 390 days post hatching. Different letters at each time point show significant differences at *P* < 0.05

The BL and BW of the genotypic triploid females, sex-reversal phenotypic triploid males and normal diploids had been analyzed and shown in Fig. 3. A rapid growth on BL and BW of yellow drum was determined from 75 to 180 dph, and a slow growth was determined from 180 to 390 dph. The BL of triploid fish was significantly higher than that of diploid fish from 75 to 150 dph. However, no significant differences were observed from 150 to 390 dph (Fig. 3A). In terms of BW, significant differences between the triploid and diploid fish were observed from 75 to 300 dph (Fig. 3B). Similar values of BW were observed from 300 to 390 dph (Fig. 3B).

### GSI and external morphology of triploid yellow drum gonads

**Fig.4:**
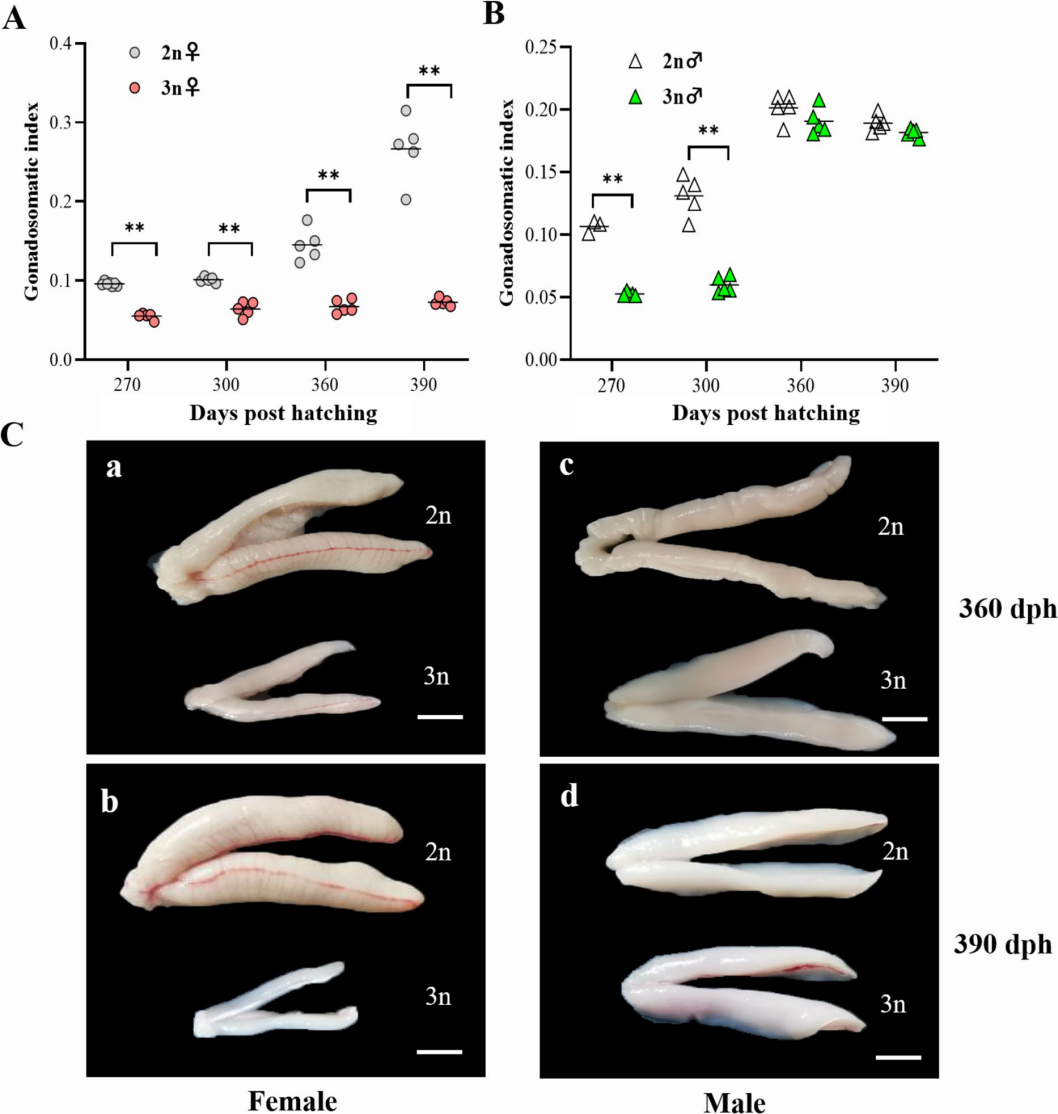
Gonadosomatic index (GSI) and external morphology of diploid (2n) and triploid (3n) yellow drums. In females, the GSI of triploid females maintained small and significantly lower than that of triploid females (**A**). In males, the GSI of triploids was significantly lower than that of diploids at 270 and 300 dph, while no significant differences in GSI at 360 and 390 dph (**B**). External features of gonads in diploid and triploid yellow drum (**C**): female at 360 dph (**a**), female at 390 dph (**b**), male at 360 dph (**c**) and male at 390 dph (**d**). Double asterisks indicate significant differences between the two groups (*P* < 0.01). Scale bars = 1.5 cm (**a**, **b**), 0.9 cm (**c**, **d**). dph, days post hatching

The GSI and external morphology of diploid and triploid gonads were analyzed and summarized in Fig. 4. In females, the GSI of diploid females was increased and significantly higher than that of triploid females from 270 to 390 dph (Fig. 4A). However, the triploid females had low GSI and small ovaries during these periods including the first spawning season (from 360 to 390 dph) (Fig. 4A and Cab). This data suggested that oogenesis was impaired in triploid females. In males, the GSI of triploids was significantly lower than that of diploids at 270 and 300 dph (Fig. 4B). While no significant differences in GSI and external morphology of testes were observed between diploid and triploid males at 360 and 390 dph (Fig. 4A and Ccd). Meanwhile, no abnormalities in external morphology were observed in triploid testes.

### Gonadal histology of triploid yellow drum

**Fig. 5:**
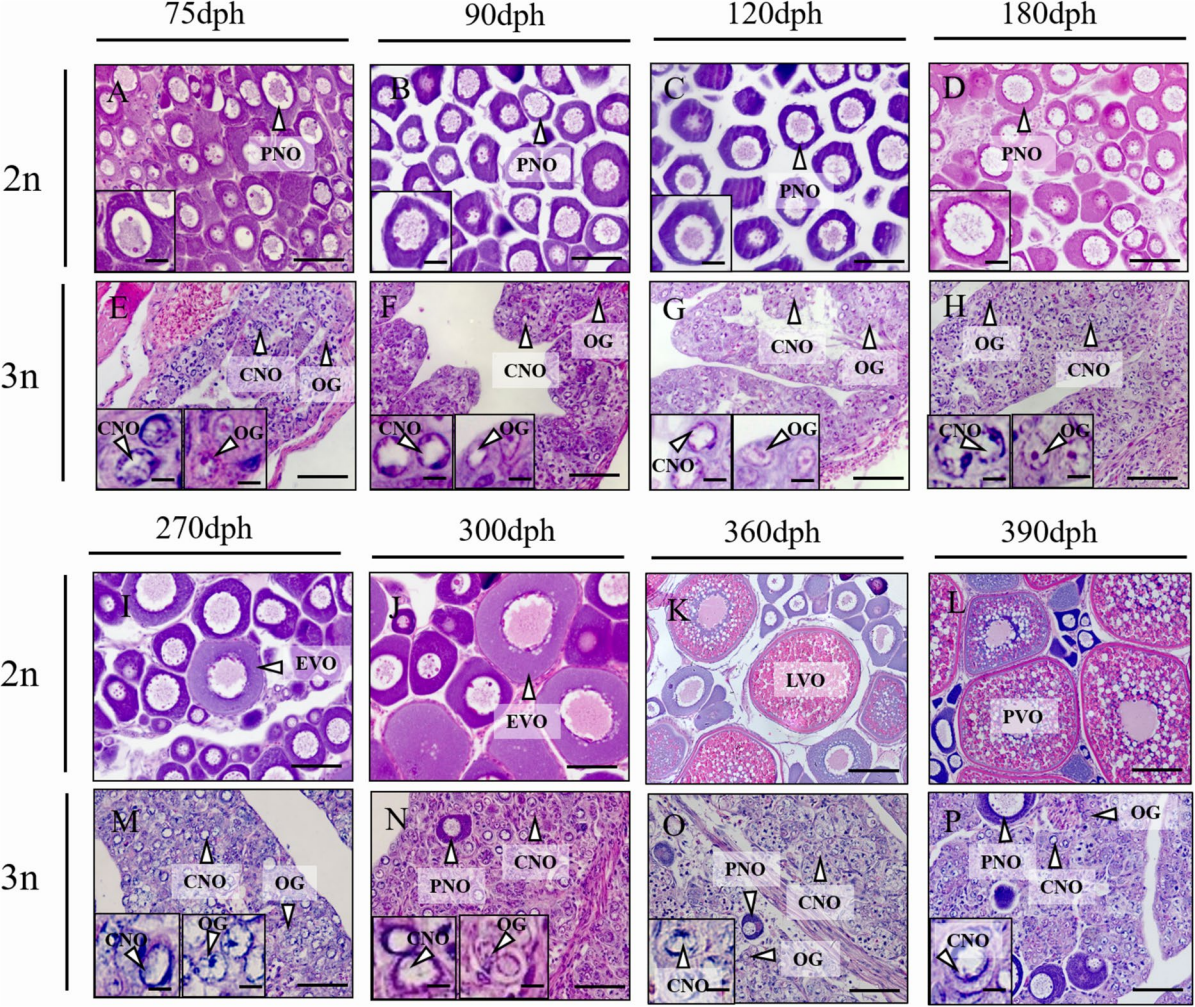
Ovarian histology of diploid (2n) and triploid (3n) yellow drum sampled at 75 (**A**, **E**), 90 (**B**, **F**), 120 (**C**, **G**), 180 (**D**, **H**), 270 (**I**, **M**), 300 (**J**, **N**), 360 (**K**, **O**) and 390 (**L**, **P**) dph. The insets indicate the magnification of main germ cell types. The insets of E–H and M-P show the CNO with chromatin adjacent to the nuclear edge and OG. OG, oogonia; PNO, perinucleolar oocyte; CNO, chromatin-nucleus oocyte; EVO, previtellogenic oocyte; PVO, postvitellogenic oocyte; LVO, late vitellogenic oocyte; dph, days post hatching. Scale bars = 80 μm (**A**-**J**, **M**-**P**), 100 μm (**K**), 250 μm (**L**), 20 μm (insert in **A**-**D**), 8 μm (insert in **E**–**H**, **M**-**P**)

The ovaries of diploid females consisted of perinucleolus stage oocytes (PNOs; Fig. 5A-D) from 75 to 180 dph. Furthermore, previtellogenic oocytes (EVOs) were observed in the ovaries of diploid females at 270 dph (Fig. 5I). However, only oogonia (OG) and chromatin-nucleolus stage oocytes (CNOs) were observed in the ovaries of triploid females from 75 to 270 dph (Fig. 5E-H, M). At 360 dph, late vitellogenic oocytes (LVOs) began appeared in diploid ovaries (Fig. 5K). Meanwhile, during the first spawning season (390 dph), many post vitellogenic oocytes (PVOs) were observed in diploid ovaries (Fig. 5L). While only a few scattered PNOs and a large number of CNOs were observed in triploid ovaries (Fig. 5N-P) from 300 to 390 dph. These data suggested that oogenesis was impaired in triploid females.

**Fig. 6:**
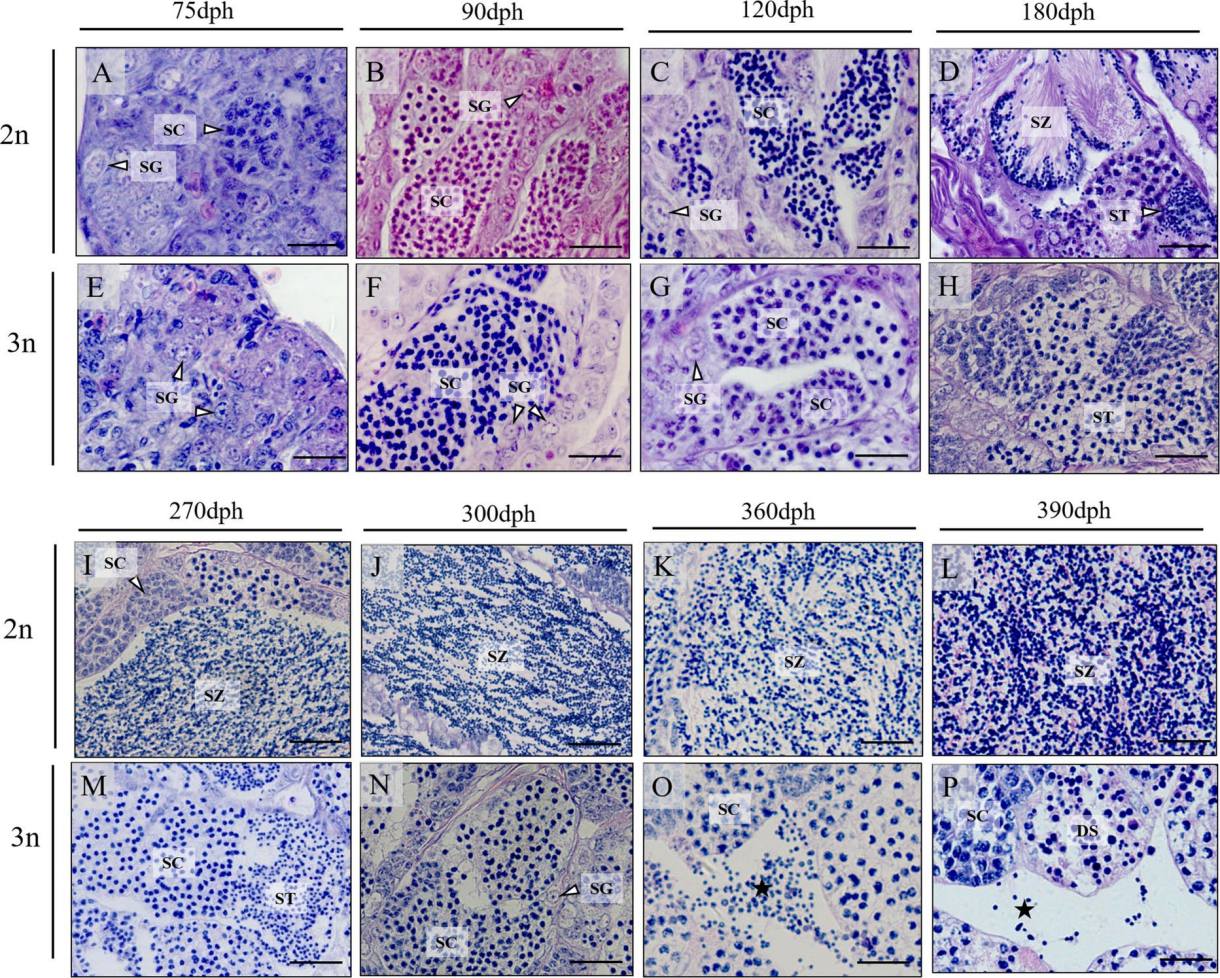
Testicular histology of diploid (2n) and triploid (3n) yellow drum sampled at 75 (**A**, **E**), 90 (**B**, **F**), 120 (**C**, **G**), 180 (**D**, **H**), 270 (**I**, **M**), 300 (**J**, **N**), 360 (**K**, **O**) and 390 (**L**, **P**) dph. Asterisk indicates SZ-like cells. DS showed highly condensed nuclei and were presumed to be undergoing apoptosis. DS, degenerated spermatocytes; SC, spermatocyte; SG, spermatogonia; ST, spermatid; SZ, spermatozoa; dph, days post hatching. Scale bars = 25 μm (**A**-**H**, **K**-**L**, **O**-**P**), 40 μm (**I**-**J**), 30 μm (**M**–**N**)

The testes of both diploid and triploid males at 75 dph were mainly composed of spermatogonia (SG; Fig. 6A and E). Furthermore, many cysts containing spermatocytes (SCs) were observed in both diploid and triploid testes at 90 and 120 dph (Fig. 6B-C and F-G). From 180 to 390 dph, the testes of diploid fish were composed of lobules filled with spermatozoon (SZ) (Fig. 6D and I-L); however, triploid testes consisted of cysts filled with either SC and spermatids (ST) (Fig. 6N-P), although a few SZ-like cells (indicated by the asterisk in Fig. 6O-P) were observed at 360 and 390 dph. Meanwhile, degenerated spermatocytes (DS) varied in size were observed in the triploid testes (Fig. 6P). DS showed highly condensed nuclei and were presumed to be undergoing apoptosis.

### Ultrastructure of germ cells in triploid yellow drum

**Fig. 7:**
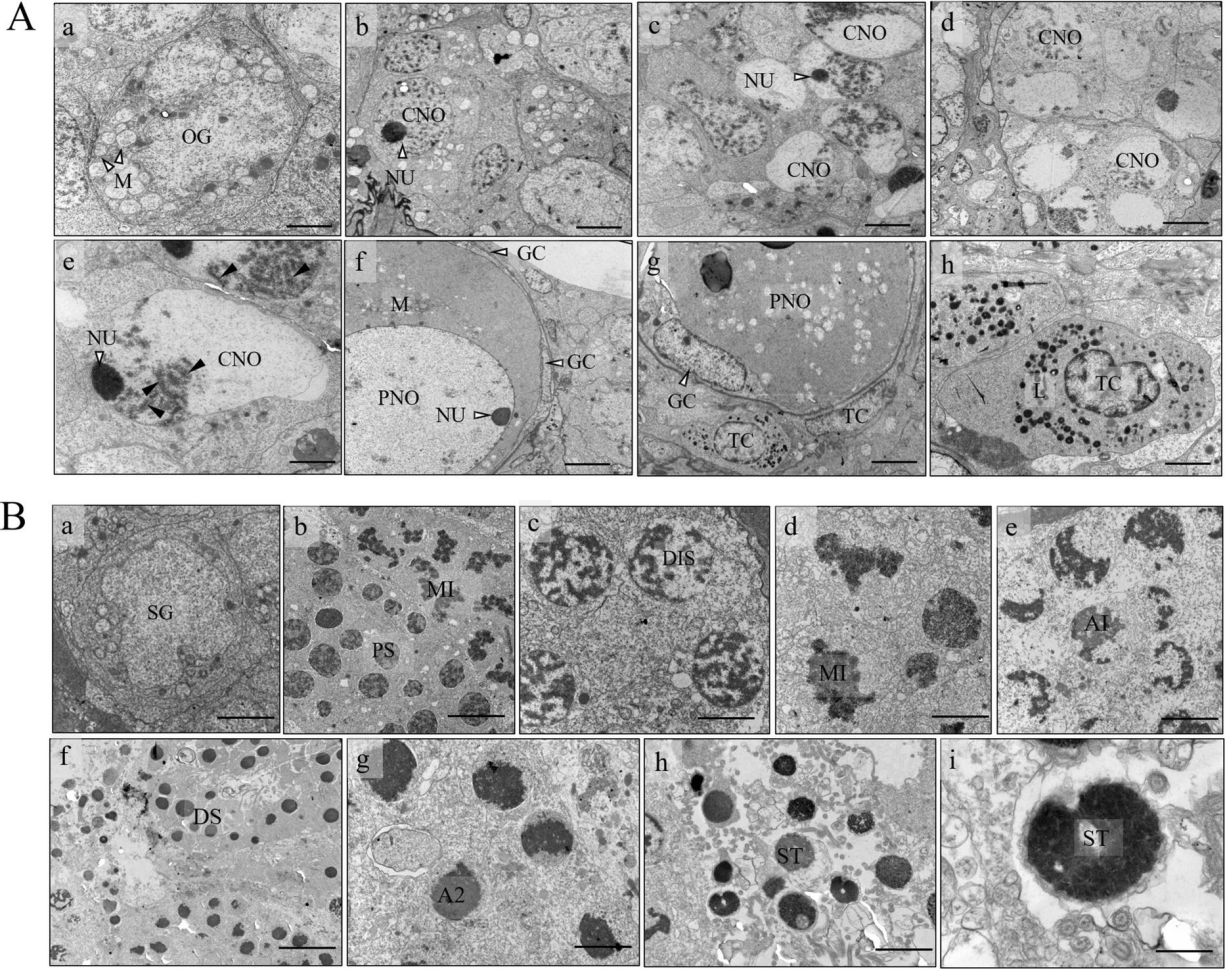
Ultrastructure of gonads in triploid female (**A**) and male (**B**) yellow drums at 360 and 390 dph. OG, oogonia; CNO, chromatin-nucleus oocyte; PNO, perinucleolus oocyte; NU, nucleous; GC, granulosa cell; TC, thecal cell; L, lysosome; M, mitochondria; SG, spermatogonia; PS, pachytene spermatocyte; DIS, diplotene spermatocytes; MI; first meiotic division; A1, anaphase/telophase 1; A2, anaphase/telophase 2; DS, degenerated spermatocyte; ST,  spermatid; dph, days post hatching. Scale bars = 2 μm (**a**, **h**), 5 μm (**b**-**d**, **g**), 2.5 μm (**e**), 4 μm (**f**) in A; 3 μm (**a**), 2.5 μm (**b**), 4 μm (**c**-**e**, **g**), 10 μm (**f**), 5 μm (**h**); 900 nm (**i**) in B

In triploid ovaries, three types of female germ cells including OG, CNO, and PNO were observed. The OG as a single isolated germ cell (approximately 10 μm in diameter) had a prominent nucleus (Fig. 7Aa). Meanwhile, some mitochondria in clusters were observed in the cytoplasm of OG. With duplication, the OG developed into CNO, which was often grouped in cysts (Fig. 7Ab-d). The CNO had a clear nucleus with a prominent nucleolus (NU) adjacent to the nuclear edge (Fig. 7Ab-e). It is worth mentioning that a number of high density and coarse chromatin material with synaptonemal complexes were observed in the NU of CNO (Fig. 7Ae). PNO contained numerous ovoid mitochondria in the cytoplasm and was surrounded by two layers of somatic cells, including the inner granulosa cell (GC) and outer thecal cell (TC) (Fig. 7Af-g). A great number of lysosomes were observed in the cytoplasm of TC (Fig. 7Ah).

In triploid testes, seven types of male germ cells including SG, five types of SC and spermatid ST were observed (Fig. 7B). The isolated SG was the largest germ cell of spermatogenic lineage (10 μm in diameter). The SG was clearly characterized by its elliptical shape, voluminous nucleus and distinctive nucleolus (Fig. 7Ba). Spermatocytes included five different stages of spermatocytes: pachytene spermatocytes (PS), diplotene spermatocytes (DIS), spermatocytes undergoing first meiotic division (MI), anaphase/telophase 1 (A1), and anaphase/telophase 2 (A2) (Fig. 7Bb-g). A number of abnormal A2 and S varied in size were observed in triploid testes (Fig. 7Bf-g). Meanwhile, the sizes of NU of abnormal S were also variable (Fig. 7Bh). These results suggested the process of sperm maturation was impaired in triploid males.

### Locations of Vasa in gonads of triploid yellow drum

**Fig. 8:**
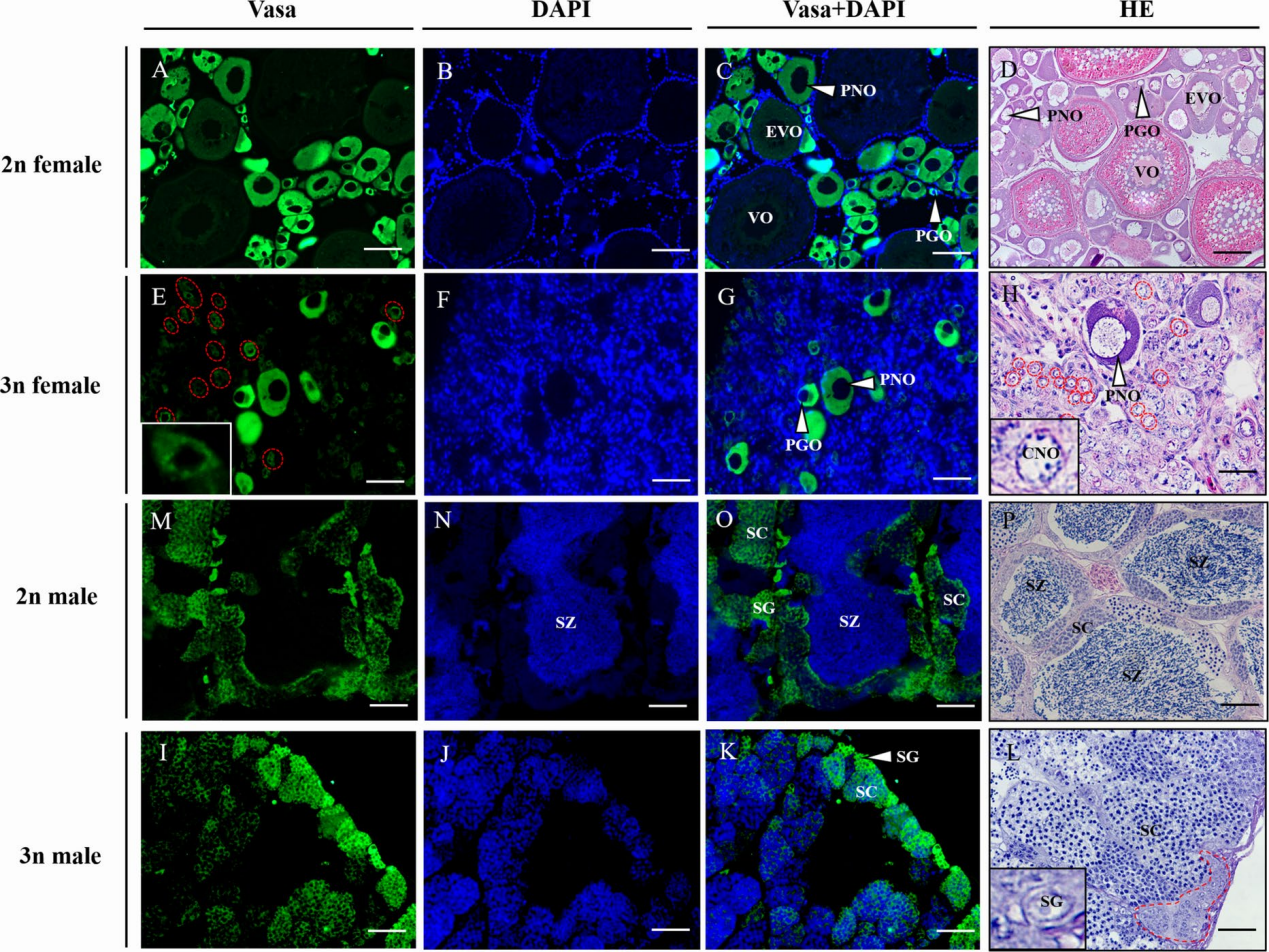
Location of Vasa protein in the gonads of diploid (2n) and triploid (3n) yellow drum by fluorescence immunohistochemistry. **D**, **H**, **P** and L are histological images stained by HE. The insets indicate the magnification of CNO in **E** and **H**. The red circles indicate the CNO. The inset shows the magnification of SG in **I**. PGO, primary growth oocyte; PNO, perinucleolus oocyte; EVO, previtellogenic oocyte; VO, vitellogenic oocyte; EVO, previtellogenic oocyte; CNO, chromatin-nucleus oocyte; SC, spermatocyte; SZ, spermatozoa; SG, spermatogonia; dph, days post hatching. Scale bars = 150 μm (**A**-**D**), 80 μm (**E**–**G**, **I**-**L**), 100 μm (M-P)

The locations of Vasa in gonads of triploid and diploid fish at 360 dph were detected (Fig. 8). In triploid and diploid ovaries, Vasa protein was cytoplasmic and mainly expressed in PNOs and PGOs (Fig. 8E-H). Meanwhile, weaker signals of Vasa protein were also detected in CNOs in triploid ovaries (Fig. 8E and H). In diploid males, Vasa protein expressed in SG and SCs in the whole testes (Fig. 8M-P). However, in triploid testes, Vasa protein was mainly expressed in SG located at the edge of gonads (Fig. 8I-L).

### Expression patterns of gonad-specific genes in triploid yellow drum

**Fig. 9:**
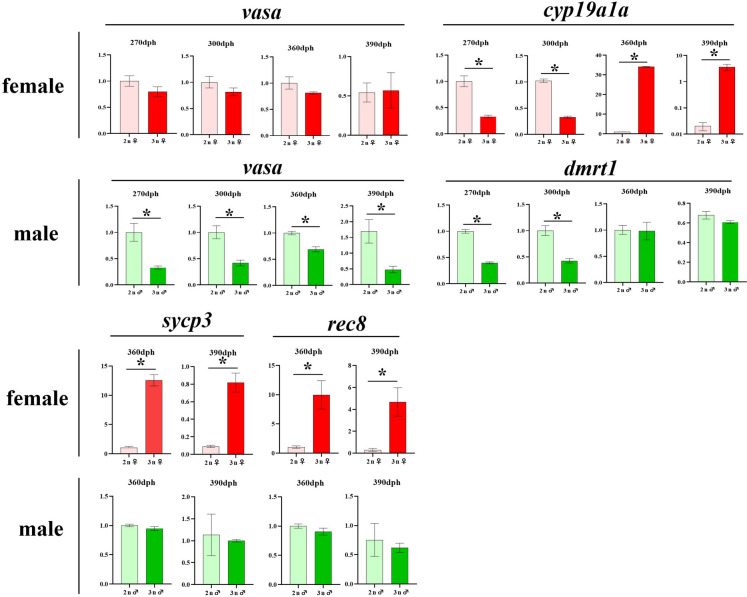
Relative expression of *vasa*, *cyp19a1a*, *dmrt1*, *sycp3* and *rec8* in the gonads of diploid (2n) and triploid (3n) yellow drum. Asterisks indicate significant differences between the two groups (*P* < 0.05)

At 270, 300, 360 and 390 dph, the expression patterns for gonad-specific genes including *vasa* and *cyp19a1a* in ovaries, and *vasa* and *dmrt1* in testes were examined. At the same time, the relative abundances of meiosis-related genes (*rec8* and *sycp3*) in gonads of both sexes at 300 and 360 dph were also examined. As shown in Fig. 9, the expression levels of germ cell marker gene, *vasa*, were similar in diploid and triploid ovaries. The expression of the female-specific gene, *cyp19a1a*, were significantly higher in diploid ovaries than that in triploid ovaries at 270 and 300 dph. On the contrary, at 360 and 390 dph, the lower expression levels of *cyp19a1a* were detected in diploid ovaries compared with sterile triploids. The meiosis-related genes, *sycp3* and *rec8* had higher expression levels in triploid ovaries than that in diploids.

In testes, *vasa* mRNA levels were significant lower in the triploid fish compared with diploids. The expression levels of the male-specific gene, *dmrt1*, were significantly higher in diploid fish than that in triploid fish at 270 and 300 dph. However, at 360 and 390 dph, no significant differences of *dmrt1* expression levels were detected between diploid and triploid testes. Meanwhile, there also were no significant differences of *sycp3* and *rec8* expression levels between diploid and triploid males.

## Discussion

In this study, we successfully produced mono-sex triploid yellow drum, including genotypic female triploids (XXX female) and sex-reversed phenotypic male triploids (XXX male). Firstly, the mono-female triploids were produced through cold-shock treatment on eggs fertilized with sperm from neo-males. Then, the mono-male triploids were produced by 100% sex reversal of mono-female triploids with oral administration of LZ. The growth performance and gonadal development in both the genotypic triploid females and sex-reversed phenotypic males during their first reproductive cycle were characterized. The results showed that triploids had similar growth performance to diploids during the first year. However, the gonadal development in both triploid females and sex-reversed males were impaired, with abnormal meiosis result in functional sterility. This study demonstrates an efficient protocol for producing sterile mono-sex triploid yellow drum.

In this study, we produced genotypic triploid females of yellow drum by cold-shock treatment of eggs fertilized with milt from neo-males. We confirmed that genotypic triploid females were 100% female by histological examinations. Further, we produced sex-reversed phenotypic triploid males by treating genotypic triploid females with oral administration of LZ for 60 days (from 30 to 90 dph) in this study. LZ is a triazole-containing aromatase inhibitor and has been used to inducing masculinization in fish, for example zebrafish (*Danio rerio*) (Uchida et al. 2004), rainbow trout (Xu et al. 2021), blue drum (*Nibea mitsukurii*) (Qin et al. 2019), diploid yellow drum (Qin et al. 2019) and yellow catfish (*Pelteobagrus fulvidraco*) (Shen et al. 2015; Yang et al. 2018). The sex-reversal phenotypic triploid males were also confirmed as 100% male by histological examinations. To the best of our knowledge, this is the first study to develop an effective method to produce genotypic triploid females and sex-reversal phenotypic triploid males in Teleostei.

Furthermore, we systematically investigated the growth performance and gonadal development of genotypic triploid females and sex-reversal males during their first year (from 75 to 390 dph). In teleosts, numerous studies have evaluated and compared the growth competences of triploids and diploids. A common thought has been acceptable that triploid fish should grow faster than their diploid counterpart, due to the reallocation of energy costs from gonadal to somatic growth in sterile triploids. However, to date, the literature on the growth rates of triploid fish is inconclusive, as triploid fish would grow faster (Qin et al. 1998; Okomoda et al. 2020) or slower (Felip et al. 2001; Ottera et al. 2016) than diploid fish. In this study, we found that the BL and BW of the triploid fish was significantly higher than that of diploid fish during the early growth period (from 75 to 180 dph). However, during the later growth period, there was no significant differences of BL and BW between triploid and diploid fish. The not-remarkable growth performance is consistent with the findings in sea bass (Felip et al. 2001) and Nile tilapia (*Oreochromis niloticus L*.) (Jamjun and Amararatne 2004). It seems that the advantage of growth of triploid fish is species-specific. However, it is worth mentioning that contradictory results of growth rates of triploid fishes between individuals of the same species have also been reported in Atlantic salmon *Salmo salar* (Maxime 2010). It is suggested that the growth differences between diploid and triploid fish are highly variable and closely dependent on the experimental conditions. Therefore, further studies of growth performance in triploid yellow drums are required.

In the present study, we observed impaired gonadal development in both the genotypic triploid females and sex-reversal males. In females, obvious smaller ovaries were observed in triploids compared to that of diploids, and no seasonal changes in the ovarian GSI of triploids were observed during the first spawning season. Histologically, although several scattered PNOs were observed, the ovaries of triploids were composed of a large number of CNOs. In teleosts, ultrastructural features of normal oogenesis are similar, the germ cells can be developed from oogonia into mature eggs in Bluefin Tuna (*Thunnus thynnus*) (Abascal and Medina 2005) and silver pomfret (*Pampus argenteus*) (Yang et al. 2021a, b). In this study, ultrastructure of germ cells showed only three types of germ cells including the OG, CNOs, and PNOs existed in triploid ovaries. Meanwhile, a number of high density and coarse chromatin material with synaptonemal complexes were observed in the NU adjacent to the nuclear edge of the CNOs. Furthermore, meiosis-related genes (*rec8* and *sycp3*) expression levels in triploid females were significantly higher than those of diploid females. These results above suggested that the germ cells in the genotypic triploid females were abnormal and retained at metaphase 1. In males, the GSI of triploids was significantly lower than that of diploids during the early development period, while no significant differences in GSI and external morphology of testes were observed between diploid and triploid males during the first spawning season. Histologically, meiosis and a delay in germ cell development were observed in sex-reversed phenotypic triploid males. However, only small amounts of SZ-like cells were produced in the lobules in triploid testes during the first spawning season. Compared with ultrastructural features of normal spermatogenesis in fish species (Yang et al. 2021a, b; Liu et al. 2021; Papah et al. 2013), triploid male yellow drum also had seven types of male germ cells including SPG, five types of SCs and SZ. However, a large number abnormal anaphase/telophase II SCs and ST varied in size and DSs were also observed in triploid testes. Furthermore, the expression levels of *sycp3* and *rec8* were similar in diploid and triploid males, implying that partial spermatogenic cells of triploid male can enter meiosis but few ST could be produced. These sterile and abnormal gonadal development are consistent with previous reports on triploid males, such as crucian carp (Zhang et al. 2021) and rainbow trout (Han et al. 2010), Nibe croaker (Takeuchi et al. 2018), grass puffer (*Takifugu niphobles*) (Hamasaki et al. 2013), and tilapia (Okomoda et al. 2020). The mechanism underlying this phenomenon is not clear, but the findings in this study strongly indicate that abnormal meiosis may result in sterile triploid females and males.

Further, we investigated the expression of gonad-specific genes in sterile triploid females and males in this study. *vasa* has been demonstrated a germ cell marker and displayed differential expression during oogenesis and spermatogenesis in teleost (Chen et al. 2005; Yoshizaki et al. 2000). Generally, Vasa (RNA and protein) is most abundant with even distribution in the cytoplasm of the spermatogonia, primary spermatocytes and oocytes at early stages in fish (Chen et al. 2005; Yoshizaki et al. 2000). In the present study, the Vasa protein abundantly expressed in PNOs and PGOs of diploid ovaries, however in sterile triploid ovaries, signals of Vasa protein were detected in a few PNOs and PGOs, and a large number of CNOs. This result exhibited abnormal gonadal development in triploid females. On the other hand, in diploid and triploid ovaries of yellow drum, the *vasa* mRNA expression levels were similar, which was in accordance with the high *vasa* expression in triploid hybrids (red crucian carp X common carp) (Yu et al. 2015). In testes, Vasa protein expressed in SG and SCs in the whole testes of diploid fish. However, in triploid testes, Vasa protein was mainly expressed in SG located at the edge of gonads. Further, the *vasa* mRNA expression levels were lower in triploids than in fertile diploid fish during both the non-breeding season and breeding season. Previous study has also shown that *vasa* expression is significantly reduced in triploid males (Yu et al. 2015). Thus, our results indicates that triploid sterility might be related to *vasa* mRNA and protein expression levels.

Sex steroid hormones produced by gonadal somatic cells play essential roles in germ cell development. It is well known that *cyp19a1a,* a female-specific gene, regulates estrogen and aromatase production during oogenesis (Xu et al. 2018). In this study, *cyp19a1a* was highly expressed in triploid ovaries during the first spawning season, although low expression levels of *cyp19a1a* were detected in sterile triploid ovaries during early developmental period (at 270 and 300 dph). In general, triploid females are known to exhibit low levels of sex steroids. For example, the low plasma levels of E2 were detected in triploid brook trout (*Salvelinus fontinalis*) (Schafhauser-Smith and Benfey 2001) and catfish (*Heteropneustes fossilis*) (Tiwary et al. 2001). However, in triploid grass puffer (Hamasaki et al. 2013) and olive flounder (*Paralichthys olivaceus*) (Wu et al. 2023) females, the plasma levels of E2 were similar to those of diploids. It is worth pointing out that the gonadal E2 levels in triploid females of olive flounder were lower than those of the diploids (Wu et al. 2023). It seems that the levels of E2 in triploid females are species-specific. In fish, oogenesis especially vitellogenesis occurs in synchrony with the increase of plasm E2 level. However, in this study, the impaired oogenesis indicated there may be a low plasm level of E2. Therefore, we speculated that the plasma E2 production could be influenced by other factor except the *cyp19a1a* in triploid yellow drum. The plasma E2 and the genes of steroidogenesis will be studied in triploids in future. In a sense, the high *cyp19a1a* expression levels indicated the triploid ovarian follicles possess the potency to produce sufficient sex steroids for oogenesis. Another sex-related gene, *dmrt1*, is known to be a key determinant of testicular development in vertebrates (Adolfi et al. 2015; Marchand et al. 2000; Webster et al. 2017). In this study, although the levels of *dmrt1* expression were lower in triploid males during early testicular development (270 and 300 dph), the *dmrt1* expression levels were similar to those of diploids during the first spawning season (360 and 390 dph). The high *dmrt1* expression levels further confirmed the masculinization and testicular development of genotypic female triploids (XXX, female) by LZ at the molecular level. In a word, those results demonstrated that the triploid yellow drum had partially functional ovarian and testicular development. This is an important characteristic as surrogate recipients used for germ cell transplantation. In this study, the aim of inducing genotypic triploid females and sex-reversal phenotypic triploid males is finally to conduct germ cell transplantation. The induction of sterile mono-sex triploids is the first step. The second step is the donor fish’s germ cells transplantation into those triploids to ultimately produce donor fish’s gametes on a large scale.

In conclusion, we successfully produced genotypic female and sex-reversed phenotypic male triploid yellow drum through the cold-shock treatment and oral administration of LZ. The gonadal development in both the triploid females and sex-reversed males were impaired, without mature eggs or sperm production during their first spawning season. Meanwhile, abnormal meiosis might lead to sterility in triploid yellow drum. Besides, the sex steroid hormones production might be at least partially functional in triploid yellow drum. These results showed that genotypic triploid females and sex-reversed phenotypic triploid males of yellow drum produced in this study have the potential for sustainable aquaculture and were suitable for utilization as surrogate recipients in germ cell transplantation in Sciaeniadae.

## Data Availability

No data was used for the research described in the article.
